# Interdialytic Weight Gain and Cardiovascular Risk in Haemodialysis Patients With Chronic Kidney Disease: Findings From a Prospective Cohort Study

**DOI:** 10.7759/cureus.97495

**Published:** 2025-11-22

**Authors:** Kavya Rajendran, Prasobh P V Mukundan, Harrie Toms John, Sheela M Chakravarthy, Rajanna Sreedhara

**Affiliations:** 1 General Medicine, Epsom and St Helier University Hospitals, London, GBR; 2 Anaesthesia, Epsom and St Helier University Hospitals, London, GBR; 3 Critical Care Medicine, Epsom and St Helier University Hospitals, London, GBR; 4 Internal Medicine, Fortis Hospital, Bangalore, IND; 5 Nephrology, Fortis Hospital, Bangalore, IND

**Keywords:** cardiovascular events, chronic kidney disease, haemodialysis, interdialytic weight gain, left ventricular hypertrophy, mortality

## Abstract

Background: Cardiovascular disease (CVD) is a major cause of morbidity and mortality in patients receiving maintenance haemodialysis (MHD). This study assessed the frequency of cardiovascular (CV) events, the factors associated with them, and the impact of interdialytic weight gain (IDWG) on clinical outcomes.

Materials and methods: A prospective cohort study was conducted at Fortis Hospital, Bangalore, India, involving 100 patients with chronic kidney disease (CKD) receiving MHD. Participants were followed for 18 months, and CV events, including myocardial infarction (MI), sudden cardiac death (SCD), atrial fibrillation (AF), and cerebrovascular accidents (CVA), were documented. IDWG was recorded at each dialysis session. Statistical analyses were performed using appropriate comparative tests for categorical variables and survival analysis methods.

Results: CV events occurred in 23% of patients, with hypertension (HTN) (p = 0.012), left ventricular hypertrophy (LVH) (p = 0.001), and IDWG > 3 kg (p = 0.015) significantly associated with increased risk. The overall mortality was 16%, mostly from cardiovascular causes.

Conclusions: IDWG > 3 kg and LVH were major predictors of adverse outcomes. Optimising fluid balance and blood pressure control may reduce cardiovascular morbidity in this population.

## Introduction

Individuals with an estimated glomerular filtration rate (eGFR) below 60 mL/min/1.73 m² face a higher likelihood of cardiovascular (CV) complications associated with chronic kidney disease (CKD). The significantly increased risk of premature mortality in patients with end-stage renal disease (ESRD) is primarily driven by CV complications [1].

Premature mortality in end-stage renal disease (ESRD) is driven predominantly by CV complications, with non-atherosclerotic cardiovascular disease (CVD) increasingly surpassing atherosclerotic manifestations in prevalence as kidney function worsens [2]. Although coronary artery disease (CAD) is common among patients with CKD, acute myocardial infarction (MI) accounts for only 14% of deaths, whereas arrhythmias contribute to approximately 66% of fatalities, according to the US Renal Data System [3].

Left ventricular hypertrophy (LVH) is present in up to 75%-85% of individuals undergoing dialysis, and hypertension (HTN) is highly prevalent in this population, contributing significantly to structural cardiac remodelling [4]. Echocardiographic (ECHO) studies demonstrate that heart failure with preserved ejection fraction (HFpEF) occurs in 85%-90% of patients with CKD and is associated with circulatory congestion, diastolic dysfunction, and recurrent fluid overload events during dialysis [2].

Patients undergoing haemodialysis (HD) for ESRD have a cardiovascular mortality rate reported to be approximately 20 times higher than that of the general population, based on age-standardised epidemiological estimates that also reflect the higher burden of comorbidities such as hypertension and diabetes [5]. Hypertension and diabetes mellitus (DM), two of the most common aetiologies of CKD, play a central role in driving CV complications among dialysis-dependent patients. In this population, the most frequent and severe CV conditions include sudden cardiac death (SCD), acute coronary syndromes (ACS), heart failure, and atrial fibrillation (AF) [6]. Furthermore, patients with CKD overall exhibit a CVD risk that is estimated to be 10-200 times higher than that of age- and sex-matched individuals without CKD [2].

From a pathophysiological standpoint, CVD in CKD is shaped by structural abnormalities (such as concentric LVH and cardiac remodelling), accelerated atherosclerosis leading to ischaemic heart disease, and arteriosclerosis affecting major vessels, resulting in increased vascular stiffness [7]. Traditional CV risk factors, including hypertension, dyslipidaemia, hypervolaemia, and sympathetic overactivity, interact synergistically with uraemia-related factors, including anaemia, hyperphosphataemia, hyperparathyroidism, elevated fibroblast growth factor-23 (FGF-23), sleep apnoea, and chronic systemic inflammation [8,9]. These overlapping mechanisms make dialysis patients particularly vulnerable to haemodynamic instability and fluid-related cardiac stress.

Interdialytic weight gain (IDWG), a readily measurable marker of fluid accumulation, reflects the degree of volume overload between dialysis sessions. Excessive IDWG is known to exacerbate blood pressure variability, cardiac workload, and myocardial strain, factors strongly linked to adverse cardiovascular outcomes in the HD population. Despite its clinical relevance, the relationship between IDWG and CV complications remains incompletely characterised, especially in regional dialysis cohorts.

This study, therefore, aimed to assess the incidence of CV complications among patients undergoing HD, to evaluate the influence of IDWG on CV outcomes, and to identify additional clinical and demographic risk factors associated with cardiovascular morbidity and mortality in this population.

## Materials and methods

This prospective cohort study was conducted at Fortis Hospital, Bannerghatta, Bangalore, India, between January 2020 and June 2021, and included 100 adult patients undergoing maintenance haemodialysis (HD). The study protocol was approved by the Institutional Ethics Committee of Fortis Hospital (approval number: IEC/023/2019) and conducted in accordance with the Declaration of Helsinki. Written informed consent was obtained from all participants prior to enrolment.

Patients were enrolled consecutively as they attended the dialysis unit and screened for eligibility based on predefined inclusion and exclusion criteria (Table 1). To ensure clinical stability, only prevalent patients on maintenance HD for ≥3 months were included. All participants had arteriovenous fistulas as their vascular access; no catheters or grafts were used.

**Table 1:** Inclusion and exclusion criteria CKD: chronic kidney disease, ESRD: end-stage renal disease, AKI: acute kidney injury, MHD: maintenance haemodialysis

Inclusion criteria	Exclusion criteria
Patients with CKD stage 5 (ESRD) receiving MHD	Patients diagnosed with AKI
Age ≥ 18 years	Individuals undergoing peritoneal dialysis
On MHD for at least 3 months	-

Demographic and clinical parameters (age, sex, comorbidities, and HD frequency) were recorded at enrolment. Patients were prospectively monitored for major cardiovascular (CV) events, including myocardial infarction (MI), sudden cardiac death (SCD), atrial fibrillation (AF), peripheral vascular disease (PVD), and cerebrovascular accidents (CVA). Interdialytic weight gain (IDWG) was calculated at each dialysis session using pre- and post-dialysis body weights, with IDWG > 3 kg considered clinically significant.

Participants were followed for 18 months, with clinical data and cardiovascular outcomes recorded quarterly. Follow-up visits were scheduled at three-month intervals, during which clinical status, dialysis parameters, and cardiovascular events were systematically recorded.

Statistical analyses were performed using IBM SPSS Statistics for Windows version 22.0 (IBM Corp., Armonk, NY). Continuous variables were expressed as mean ± standard deviation (SD). Associations between categorical variables, including comorbidities, symptoms, medication use, and cardiovascular outcomes, were assessed using the Chi-square test. Survival and cardiovascular event-free survival were evaluated using the Kaplan-Meier method, and group differences were compared using the log-rank test. The Cochran-Armitage trend test was applied to assess linear associations between IDWG categories and cardiovascular outcomes. A p-value of <0.05 was considered statistically significant.

Owing to the relatively small number of cardiovascular events and incomplete confounder data, multivariate regression and Cox proportional hazards analyses were not feasible. This limitation was recognised and transparently stated in the Discussion.

## Results

Of the 100 individuals initially assessed for eligibility, 100 participants were included in the study based on the predefined inclusion criteria.

Patient demographics and clinical characteristics

The study included 100 adults receiving maintenance haemodialysis (MHD), with a mean age of 64 ± 10 years and a male-to-female ratio of roughly 3:2. Most participants (95%) underwent thrice-weekly dialysis. Hypertension and diabetes mellitus were the predominant comorbidities, while previous myocardial infarction, cerebrovascular accident, and hypothyroidism were less frequent. Dyspnoea on exertion was the most common symptom, and only a few patients reported orthopnoea or palpitations. The majority were prescribed beta-blockers, statins, or antiplatelet agents, whereas angiotensin-converting enzyme inhibitors (ACEis) or angiotensin receptor blockers (ARBs) were used infrequently. Baseline demographic and clinical characteristics are summarised in Table 2.

**Table 2:** Characteristics of the study population SD: standard deviation, DM: diabetes mellitus, HTN: hypertension, PVD: peripheral vascular disease, MI: myocardial infarction, CVA: cerebrovascular accident, ACEi: angiotensin-converting enzyme inhibitor, ARB: angiotensin II receptor blocker

Parameters	Number (100)
Baseline characteristics	
Age, years (mean ± SD)	64.28 ± 10.49
Gender, male (%)	59/41
Dialysis frequency	
Twice weekly (number (%))	5 (5)
Thrice weekly (number (%))	95 (95)
Underlying medical condition	
DM (number (%))	43 (43)
Duration of DM, years (mean ± SD)	12.21 ± 7.891
HTN (number (%))	77 (77)
Duration of HTN, years (mean ± SD)	9.95 ± 5.505
MI (number (%))	10 (10)
Duration since MI, years (mean ± SD)	2.80 ± 1.317
CVA (number (%))	3 (3)
Duration since CVA, years (mean ± SD)	7.00 ± 0
PVD (number (%))	0 (0)
Hypothyroidism (number (%))	19 (19)
Duration of hypothyroidism, years (mean ± SD)	8.29 ± 3.368
Current complaints	
Angina (number (%))	0 (0)
Dyspnoea on exertion (number (%))	20 (20)
Palpitation (number (%))	0 (0)
Orthopnoea (number (%))	2 (2)
Current medications	
ACEi/ARB (number (%))	4 (4)
Beta-blockers (number (%))	38 (38)
Aspirin/clopidogrel (number (%))	26 (26)
Statin (number (%))	32 (32)
Anti-anginal (number (%))	0 (0)

At the end of the study, 75 (75%) patients were alive, 16 (16%) died, and nine (9%) were lost to follow-up. Among the 16 deceased, 15 had experienced CV events.

Incidence of CV events during the study period

**Figure 1:**
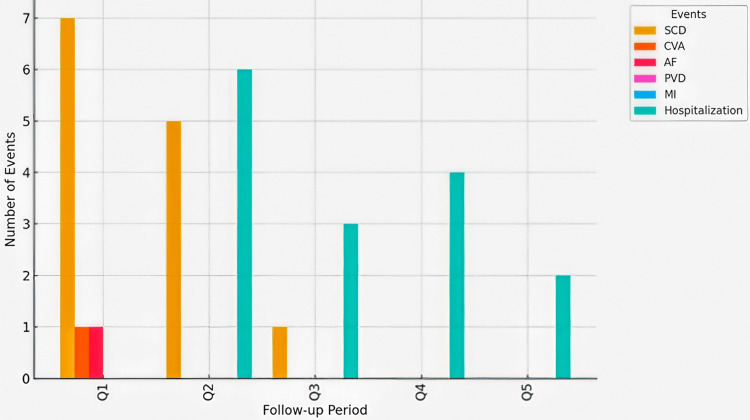
Distribution of cardiovascular events by follow-up quarter Q1-Q5: quarterly follow-up periods (18 months), SCD: sudden cardiac death, CVA: cerebrovascular accident, AF: atrial fibrillation, PVD: peripheral vascular disease, MI: myocardial infarction

A substantial concentration of CV events occurred during the first two quarters of follow-up, with 11 out of 14 myocardial infarctions (78.6%) and 12 out of 13 SCD (92.3%) recorded during this period (Figure 1). This early clustering of events suggests a phase of increased vulnerability, potentially influenced by patients’ baseline health status and external factors such as the COVID-19 pandemic.

Association between comorbidities and CV events

The results from the thorough assessment of the link between primary comorbid conditions and CV complications in patients with CKD on HD are summarised in Table 3. For this analysis, associations between categorical variables (comorbidities and cardiovascular complications) were evaluated using the Chi-square (χ²) test. The test statistics and p-values were as follows: for hypertension, χ²(1) = 6.35 and p = 0.012, and for hypothyroidism, χ²(1) = 10.54 and p = 0.001. Other comparisons, including diabetes, myocardial infarction, and cerebrovascular accident, yielded non-significant results (p > 0.05). Therefore, hypertension and hypothyroidism were significantly associated with CV events (p = 0.012 and p = 0.001, respectively). No significant associations were observed for diabetes, prior myocardial infarction, or cerebrovascular accident.

**Table 3:** Association between cardiovascular complications and comorbidities in patients with CKD undergoing HD DM: diabetes mellitus, HTN: hypertension, MI: myocardial infarction, CVA: cerebrovascular accident, PVD: peripheral vascular disease *p < 0.05: statistically significant **No patients in the study cohort were diagnosed with PVD; therefore, statistical comparison and p-value calculation were not applicable for this comorbidity

Comorbidities		Cardiovascular complications		p-value
		Yes	No	
		Number (%)	Number (%)	
DM	No	10 (17.5)	47 (82.5)	0.082
	Yes	1 (32.64)	29 (67.4)	
HTN	No	1 (4.3)	22 (95.7)	0.012*
	Yes	23 (29.9)	54 (70.1)	
MI	No	23 (25.6)	67 (74.4)	0.275
	Yes	1 (10)	9 (90)	
CVA	No	24 (24.7)	73 (75.3)	0.323
	Yes	0 (0)	3 (100)	
PVD	No	24 (24)	76 (76)	**
Hypothyroidism	No	14 (17.3)	67 (82.7)	0.001*
	Yes	10 (52.6)	9 (47.4)	

Association between symptoms on follow-up and CV complications in patients with CKD undergoing HD

There was a significant association between symptoms on follow-up (dyspnoea and orthopnoea) and CV complications. These were analysed using the Chi-square (χ²) test, which demonstrated a significant relationship (χ² = 7.05, p = 0.008). Patients reporting symptoms had a higher frequency of cardiovascular events compared with asymptomatic patients, indicating symptom burden as a potential predictor of adverse outcomes. Among the 16 patients who reported symptoms, 8 (50%) experienced CV complications, compared to 16 out of 84 (19%) patients without symptoms.

Effect of medications on CV events

The use of beta-blockers, statins, antiplatelet agents, and anti-anginal drugs showed significant associations with CV events. These were assessed using the Chi-square (χ²) test. Significant associations were observed for beta-blockers (χ² = 6.04, p = 0.014), antiplatelet agents (χ² = 7.60, p = 0.006), statins (χ² = 9.47, p = 0.002), and anti-anginals (χ² = 9.80, p = 0.002), suggesting that patients receiving these medications had higher cardiovascular complication rates. The detailed distribution of CV events by medication use is summarised in Table 4.

**Table 4:** Cardiovascular complications by medication use CV: cerebrovascular, ACEi: angiotensin-converting enzyme inhibitor *p < 0.05: statistically significant

Medication	Patients with CV complications (number (%))	Patients without CV complications (number (%))	p-value
ACEi	2 (40)	3 (60)	0.390
Beta-blockers	16 (35.6)	29 (64.4)	0.014*
Aspirin/clopidogrel	14 (40)	21 (60)	0.006*
Statins	16 (40)	24 (60)	0.002*
Anti-anginals	3 (100)	0 (0)	0.002*

Significance of interdialytic weight gain and CV events

Patients with interdialytic weight gain (IDWG) greater than 3 kg experienced a significantly higher incidence of CV events (35%) than those with IDWG below 3 kg (10%; p < 0.01). This threshold was used to define clinically relevant fluid overload. This difference was statistically significant (p < 0.01), indicating a 3.45-fold increased risk of CV complications in patients with IDWG exceeding 3 kg. To provide further clarity, CV events were also analysed after stratifying patients by IDWG categories (<2 kg, 2-3 kg, 3-4 kg, and >4 kg). The results are presented in Table 5. However, trend analysis using the Cochran-Armitage test did not demonstrate a significant linear association between increasing IDWG and CV risk (Z = 1.00, p = 0.316).

**Table 5:** Association between IDWG categories and cardiovascular complications IDWG: interdialytic weight gain, CV: cardiovascular

IDWG category	Patients (number)	Patients with CV events (number (%))	Patients without CV events (number (%))
<2 kg	10	1 (10)	9 (90)
2-3 kg	39	4 (10.3)	35 (89.7)
3-4 kg	31	11 (35.5)	20 (64.5)
>4 kg	20	7 (35)	13 (64)
Total < 3 kg	49	5 (10.2)	44 (89.8)
Total > 3 kg	51	18 (35.2)	33 (64.8)
Total	100	23 (23)	77 (77)

Electrocardiographic (ECG) and echocardiographic (ECHO) findings

Among patients with abnormal ECG findings (including LVH, T-wave inversion, or ST-segment changes), 13 out of 32 (40.6%) had CV complications, compared to 11 out of 68 (16.2%) among those with normal ECGs (p < 0.01).

Regarding ECHO findings, 16 out of 22 (72.7%) patients with concentric left ventricular hypertrophy (LVH) experienced CV complications, while only eight out of 55 (14.5%) of those with normal ECHO findings had such complications (p < 0.001).

Association between ECHO findings and CV complications

**Figure 2:**
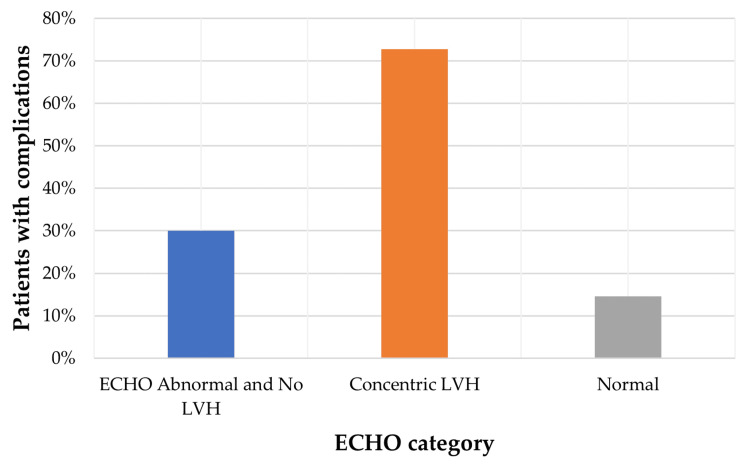
ECHO findings versus cardiovascular complications ECHO: echocardiography, LVH: left ventricular hypertrophy

Left ventricular hypertrophy was strongly associated with CV events, confirming the prognostic importance of structural cardiac abnormalities on echocardiography (χ² = 18.133, df = 2, p < 0.001). As illustrated in Figure 2, the incidence of CV complications varied notably with different ECHO patterns. Patients with LVH on ECHO showed the highest prevalence of CV events, followed by those with abnormal ECHO findings without LVH. In contrast, patients with normal ECHO findings exhibited the lowest complication rates. These results highlight the prognostic relevance of structural cardiac abnormalities identified on ECHO in this patient population.

Association between CV events and mortality in patients with CKD undergoing HD

**Figure 3:**
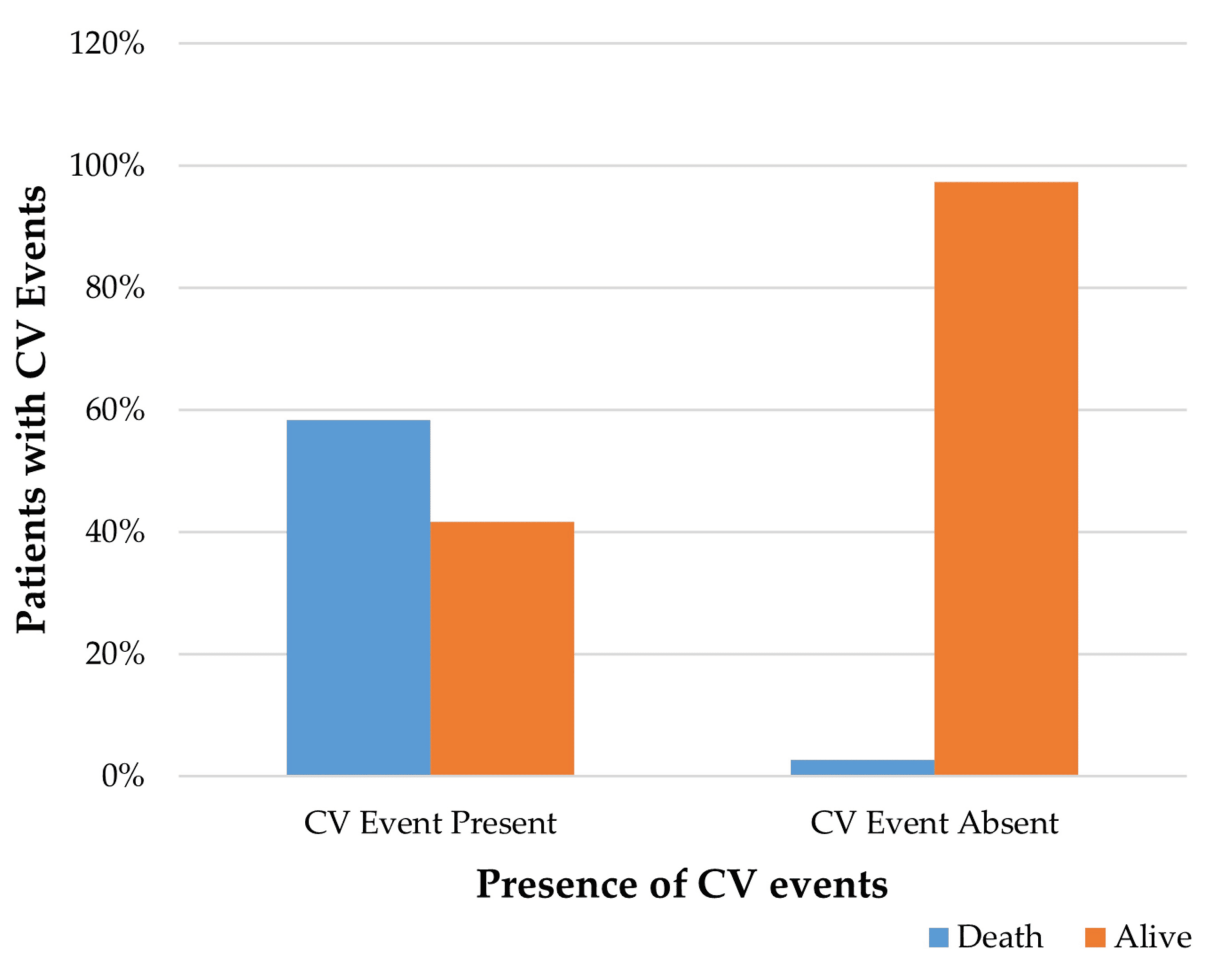
Association between CV events and patient outcomes CV: cardiovascular

There was a significant association between CV events and patient outcomes. Among individuals who experienced CV events (n = 23), 21 died by the end of follow-up. In contrast, only two deaths occurred among those without CV events (n=64). This was analysed using the Chi-square (χ²) test, which showed a statistically significant relationship (χ² = 15.24, p < 0.001). Mortality was markedly higher among patients who experienced CV events, confirming their major contribution to poor overall survival outcomes (Figure 3).

Kaplan-Meier survival analysis

**Figure 4:**
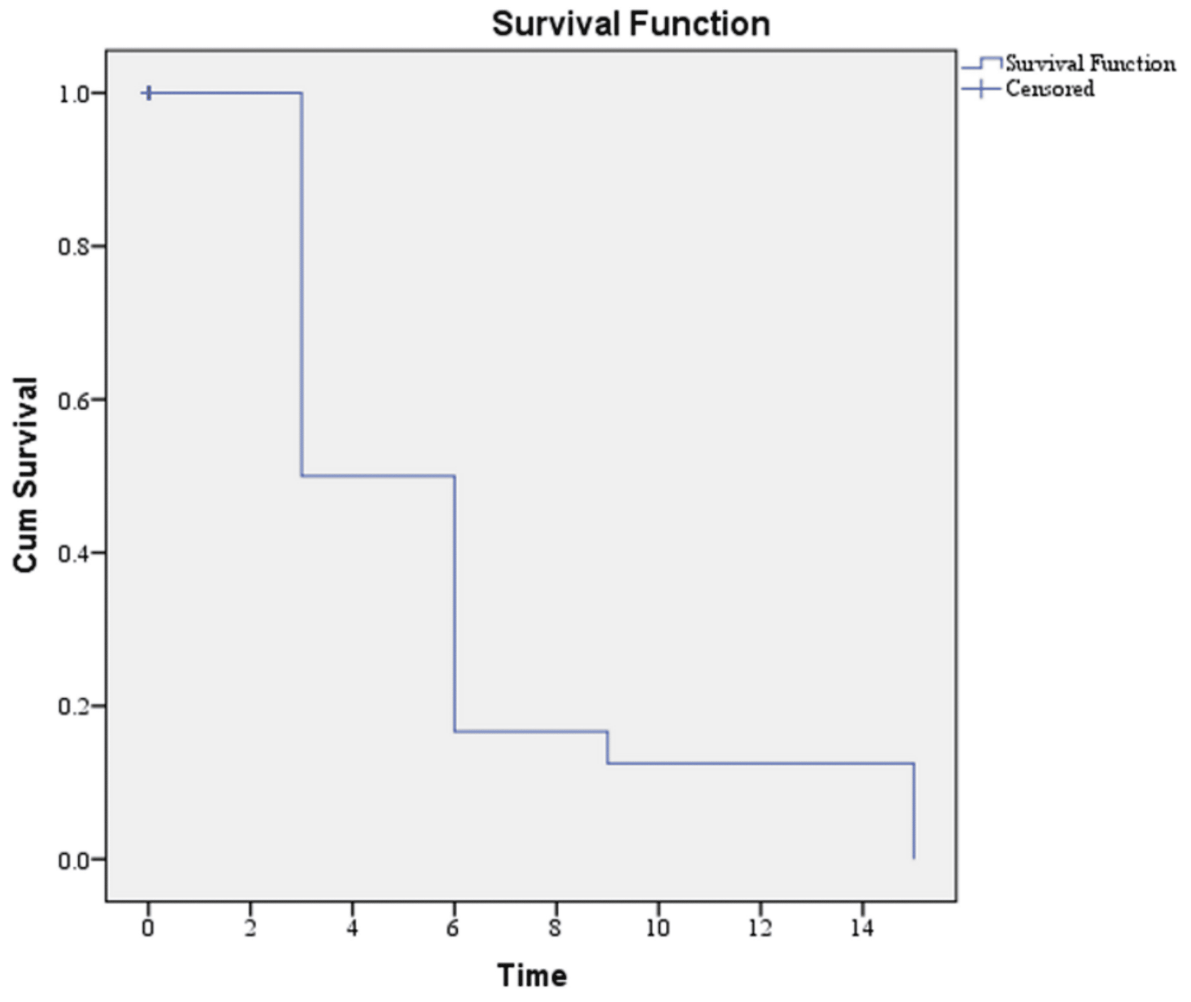
Kaplan-Meier survival curve for the studied cohort

The Kaplan-Meier survival analysis revealed a mean survival time of 7.78 ± 1.09 months and a median survival time of six months among patients who experienced CV events. The mean time to onset of CV events was 5.75 ± 0.80 months. These findings highlight the rapid deterioration following CV events in patients with CKD undergoing HD (Figure 4).

## Discussion

CKD encompasses a range of pathophysiological processes resulting from impaired kidney function and a gradual reduction in glomerular filtration rate (GFR). Its progression is primarily associated with reduced eGFR levels and the severity of albuminuria [3]. At any stage of CKD, individuals face a 10- to 200-fold higher CV risk compared to the general population matched for age and sex [2]. As a result, the majority of patients develop some form of CVD before progressing to stage 5 CKD. Among those who do reach stage 5, 45% present with advanced CV complications; therefore, early intervention is crucial to prevent cardiac complications in patients with CKD [2].

The risk factors for CVD in patients with CKD can be categorised into traditional and uraemia-related factors. Traditional risk factors include hypertension, hypervolaemia, dyslipidaemia, sympathetic overactivity, and hyperhomocysteinaemia. In contrast, uraemic risk factors comprise anaemia, hyperphosphataemia, hyperparathyroidism, elevated FGF-23 levels, sleep apnoea, and systemic inflammation [3].

ESRD, defined as a life-threatening reduction in renal function that requires dialysis or transplantation for survival, is the most visible consequence of CKD [6]. In ESRD, treatment options consist of HD, peritoneal dialysis (either as continuous ambulatory peritoneal dialysis (CAPD) or continuous cyclic peritoneal dialysis (CCPD)), and renal transplantation [3]. CVD remains the leading cause of mortality among patients with ESRD undergoing HD [2,4]. In CKD stages 2 and 3, CV mortality is primarily linked to traditional risk factors, whereas in patients with CKD stage 4 and who are dialysis-dependent, novel risk factors play a more significant role. Studies have shown that the incidence of CV events is highest in the initial weeks following HD initiation, raising concerns that the dialysis process itself may act as a trigger for such events [3]. While interdialytic weight gain and fluid overload are recognised risk factors, our study contributes prospective evidence from a South Asian HD cohort. Specifically, it validates the >3 kg threshold as clinically meaningful in predicting CV events, offering region-specific insights for practice.

Incidence of CV complications

The study of the United States Renal Data System (USRDS) DMMS Wave 2 data shows that the prevalence of CAD among patients who were initiated on dialysis in 1996/1997 was 38%. Also, in the Case Mix Severity Study, describing comorbid conditions of new patients in 1986/1987, a similar prevalence of 40% was reported [3]. However, in the present study, 14% of the patients had MI.

Incidence of SCD

The United States Renal Data System (USRDS) 2010 identified SCD as the cause of death in 25% of all fatalities among HD patients [10]. In a large, randomised trial involving 3,883 HD patients, CV causes accounted for 54% of all deaths, with SCD representing 24.5% of these cases [11]. In contrast, the incidence of SCD in the present study was notably lower, occurring in only 13% of cases.

Mortality due to CV complications in patients with CKD undergoing HD

In the present study, 16% of patients died during follow-up, with SCD accounting for 81% of these cases. This contrasts with USRDS data from 1998, which reported CVD as the cause of 40% of deaths among 335,000 patients with ESRD [2].

Most MI and SCD events occurred within the first six months of follow-up. As the study began in January 2020, during the initial wave of the COVID-19 pandemic, this timing may partly explain the increased incidence of CV events observed early in the follow-up period.

Association between comorbidities and CV complications in patients with CKD undergoing HD

The present study found no statistically significant association between DM, prior MI, or CVA and CV complications in HD patients, contrasting with previous evidence, such as Foley et al., who reported high mortality in CKD largely due to accelerated atherosclerotic vascular disease and congestive heart failure [12]. This difference may be explained by our small sample size, survivor bias from the exclusion of patients with severe CV history from long-term dialysis, and a short follow-up period insufficient to capture cumulative effects. In contrast, we observed a significant association between HTN and CV complications, consistent with reports that poorly controlled HTN contributes to LVH, arterial stiffness, and endothelial dysfunction, thereby increasing morbidity and mortality in dialysis patients [13]. In HD, fluid shifts and volume overload further destabilise blood pressure, exacerbating CV strain and accelerating disease progression. Although DM and atherosclerotic CVD are common in CKD, dialysis-related factors such as sympathetic overactivity, chronic inflammation, and vascular calcification may play a more dominant role, potentially overshadowing traditional risk factors in short-term analyses. Additionally, our finding of a significant association between hypothyroidism and CV complications aligns with Liu et al., who proposed that thyroid dysfunction in CKD can promote dyslipidaemia, endothelial dysfunction, and atherosclerosis, with these effects amplified in dialysis by altered hormone metabolism, inflammation, and fluid-electrolyte imbalances [14]. These observations highlight the importance of optimising blood pressure control and monitoring thyroid function in patients with CKD on HD to reduce CV risk.

Although LVH, atrial fibrillation, and compliance are well-established predictors of survival, our study focused on more accessible clinical variables. Future research should incorporate echocardiographic and rhythm-based assessments for more comprehensive risk profiling.

Association between cardiac symptoms and complications in patients with CKD undergoing HD

In our study, CV complications were observed in 50% of HD patients reporting cardiac symptoms during follow-up, compared with only 19% among asymptomatic individuals. This suggests that symptom presence may serve as an important early indicator of heightened CV risk in this group. Previous research has shown that in CKD and ESRD, symptoms such as dyspnoea, chest discomfort, and reduced exercise capacity often reflect structural heart changes, fluid overload, or arrhythmias, and are linked to worse outcomes. Zoccali et al. demonstrated an association between such symptoms, increased left ventricular mass, and reduced survival, highlighting the prognostic value of vigilant symptom monitoring [15].

Association between drugs and CV complications in patients with CKD undergoing HD

The present study identified significant associations between the use of beta-blockers, statins, antiplatelet agents, and anti-anginal medications and the presence of CV complications. This contrasts with findings from a cohort study conducted in Ontario, Canada, involving 1,836 patients (504 beta-blocker users, 570 calcium channel blocker users, and 762 statin-only users), where beta-blocker use did not show improved CV outcomes compared to statin-only therapy [16]. A plausible explanation for the observed association in the current study is that patients receiving these medications had already experienced prior CV events or had more advanced cardiac disease at baseline. In clinical practice, beta-blockers, antiplatelets, and anti-anginal drugs are typically prescribed in the context of established CAD, prior myocardial infarction, or symptomatic ischaemia. Thus, the presence of these medications in a patient’s therapeutic regimen often reflects the severity and chronicity of underlying CV pathology. Rather than implying a harmful effect of these agents, the association likely highlights that patients requiring such therapies are inherently at higher risk for recurrent CV complications. These findings underscore the importance of early risk stratification and secondary prevention strategies in high-risk populations.

The associations between beta-blocker, statin, and antiplatelet use and CV events likely reflect treatment indication bias, since these therapies are prescribed to patients already at higher CV risk. Thus, causality should not be inferred.

Association between ECG changes and CV complications in patients with CKD undergoing HD

A study involving 39 patients with CKD demonstrated that individuals with chronic renal failure frequently present with ECG abnormalities and have a high prevalence of arrhythmias, particularly during and after dialysis sessions [17,18]. In the present study, a significant association was found between ECG changes, including LVH, T-wave inversion, and ST-segment abnormalities, and the occurrence of CV complications.

Patients presenting with ECG abnormalities experienced a higher incidence of CV events compared to those without such findings. This highlights the potential value of routine ECG evaluation as a simple, non-invasive, and accessible tool for early identification of patients at increased CV risk in the HD population. Incorporating regular ECG monitoring into standard care protocols may support timely risk stratification and guide the implementation of preventive strategies in this high-risk group.

Association between LVH in ECHO and CV complications in patients with CKD undergoing HD

In our cohort, concentric LVH detected on echocardiography was significantly associated with CV complications in patients receiving HD, underlining its value as a prognostic marker. LVH is a frequent finding in CKD and has been consistently linked to increased CV morbidity and mortality. Foley et al. reported that LVH independently predicted both CV events and overall mortality in dialysis patients. Contributing factors include chronic volume and pressure overload, anaemia, and vascular stiffness, which drive maladaptive myocardial changes [12]. These results support the need for routine LVH assessment and targeted management strategies to improve outcomes in this high-risk group.

Significance of the association between IDWG and CV complications in patients with CKD undergoing HD

In the present study, patients with IDWG greater than 3 kg experienced over three times the risk of developing CV complications compared to those with lower IDWG. This finding is consistent with previous evidence indicating that excessive fluid retention between dialysis sessions contributes to persistent HTN, progressive LVH, and elevated CV morbidity. Kalantar-Zadeh et al. reported that higher IDWG correlates with increased rates of mortality and hospitalisation in HD populations, largely through mechanisms of chronic volume overload and HD strain [19]. These observations emphasise the critical role of strict fluid control and patient education in reducing CV risk.

However, the stratified analysis across four IDWG categories (<2 kg, 2-3 kg, 3-4 kg, and >4 kg) did not demonstrate a linear trend of progressively increasing CV risk. Instead, our data suggests the existence of a clinically meaningful threshold at >3 kg, beyond which the risk rises substantially. This highlights the value of the 3 kg cutoff as a practical target in clinical monitoring and fluid management strategies for patients undergoing HD.

Survival analysis

In our cohort of 100 HD patients, those who experienced CV events had a mean survival time of 7.78 ± 1.09 months and a median survival of six months, while the mean time to the first CV event was 5.75 ± 0.81 months. These results highlight the short interval between the onset of CV complications and death in this group. Comparable findings were described by Stack and Bloembergen, who reported that CV events are the predominant cause of mortality in HD patients, with survival significantly reduced following such events [19]. This reinforces the importance of proactive CV surveillance and early intervention to improve outcomes in high-risk individuals.

Limitations of the study

Several limitations should be acknowledged. The study period coincided with the first wave of the COVID-19 pandemic, which may have influenced access to medical care, contributed to underreporting of non-COVID complications, and affected CV outcomes. The direct effect of SARS-CoV-2 infection could not be clearly distinguished from the underlying disease burden.

Statistical analyses were restricted to univariate Chi-square tests and Kaplan-Meier survival methods; multivariate regression and Cox proportional hazards models could not be applied due to the relatively small number of CV events and incomplete confounder data.

Other limitations include the small sample size, single-centre design, and potential survivor bias, which may affect external validity. The limited number of cardiovascular events precluded multivariable adjustment for confounders; however, this was transparently acknowledged to avoid overinterpretation of associations.

IDWG was analysed as an absolute value (>3 kg) instead of being expressed relative to baseline dry weight, which may limit comparability with other studies. Iron indices (ferritin and transferrin saturation) were not consistently available and could not be analysed. As iron deficiency has been linked to increased CV morbidity in HD patients, this represents a limitation [20]. Similarly, data on parathyroid hormone (PTH), calcium, and phosphate were unavailable. Given their impact on vascular stiffness and CV morbidity, omission of these parameters is another limitation [21]. Finally, potential underreporting bias cannot be excluded, as some data were based on patient self-reports.

## Conclusions

This prospective study confirms the substantial burden of CV complications among patients with ESRD undergoing HD and identifies HTN, LVH, and IDWG > 3 kg as key predictors of morbidity and mortality. The study provides prospective, region-specific evidence from a South Asian HD cohort, confirming >3 kg IDWG as a clinically meaningful threshold for CV risk. Our data suggests that the 3 kg cutoff represents a practical clinical threshold rather than a gradual risk continuum, supporting its use in routine monitoring and patient counselling. The findings also show that CV events tend to cluster in the initial months of HD, underscoring the need for intensified surveillance during this vulnerable period. Conducted during the early COVID-19 pandemic, the study additionally reflects the heightened vulnerability of HD patients to CV complications under conditions of infectious and haemodynamic stress. Despite inherent limitations, these results highlight the importance of strict volume control, early CV risk assessment, and proactive intervention, and they support the need for larger multicentre studies to validate and extend these observations.
